# Self-care knowledge and contributing factors among ambulatory diabetes mellitus patients in North-Central, Ethiopia: a facility-based cross-sectional study

**DOI:** 10.11604/pamj.2025.52.59.40343

**Published:** 2025-10-03

**Authors:** Tomas Getahun, Woldu Gebrehiwot, Yonas Abebe, Marekegn Habtamu

**Affiliations:** 1 Armauer Hansen Research Institute, Ministry of Health, Addis Ababa, Ethiopia; 2 Department of Public Health, College of Health Science, Debre Berhan University, Debre Berhan, Ethiopia; 3 Ethiopian Field Epidemiology and Laboratory Training Program, Ministry of Health, Addis Ababa, Ethiopia; 4 Department of Human Nutrition, Menelik II College of Medicine and Health Science, Kotebe Metropolitan University, Addis Ababa, Ethiopia

**Keywords:** Diabetes mellitus, self-care knowledge, North-Central Ethiopia

## Abstract

**Introduction:**

Diabetes Mellitus (DM) is a serious, chronic medical condition. It requires ongoing medical follow-up and patient self-management. However, poor self-management and diabetes complications are the result of inadequate diabetes self-care knowledge. Hence, this study aimed to investigate diabetes self-care knowledge and associated factors among diabetes patients in North-Central, Ethiopia.

**Methods:**

a facility-based cross-sectional study design was employed among 355 diabetes patients at North-Central, Ethiopia, public and private health facilities from March 22^nd^ to April 30^th^, 2022. A systematic sampling technique was applied to select study participants. The data were collected using a structured interviewer-administered questionnaire and by reviewing patient charts. We used Epidata version 4.6 to enter the data, and SPSS version 23 was used for data analysis. Both bivariable and multivariable logistic regression were used to identify contributing factors for diabetic patients´ self-care knowledge.

**Results:**

among the total participants, 54% had good knowledge about diabetes self-care. Variables like, educational level diploma and above (AOR: 3.407, 95% CI: 1.337-8.684), monthly income of 2000 birr and above (AOR: 4.384, 95% CI: 1.548-12.411), respondents with regular follow-up (AOR: 8.910, 95% CI: 2.903-17.349), and those who have ever attended diabetes health education (AOR: 5.554, 95% CI: 1.820-16.953) were significantly associated with having good diabetes self-care knowledge.

**Conclusion:**

more than half of the participants have good knowledge of diabetes self-care. As per our results, educational level, monthly income, regular follow-up, and having experience in diabetes-related health education were independent determinants of having good knowledge about diabetes self-care.

## Introduction

Diabetes Mellitus (DM) is a serious chronic medical condition that occurs either when the pancreas fails to generate enough insulin or when the body fails to use it effectively. Therefore, clinically, it is identified as a raised level of blood glucose (hyperglycemia). DM is broadly categorized into type I, which contributes 10% of the total prevalence of the case, and type II, which is the most common type of diabetes and accounts for about 90% of the total prevalence [1]. This long-lasting metabolic disorder and uncontrolled blood sugar levels affect multiple organ systems. It can lead to serious microvascular and macrovascular complications. Microvascular complications include neuropathy, nephropathy, and retinopathy. Macrovascular complications include cardiovascular disease, stroke, and lower limb amputation [2-4].

According to the International Diabetes Federation (IDF) Diabetes Atlas, 10^th^ edition 2021 report, globally, 537 million adults aged 20-70 are living with diabetes in 2021. This is equivalent to 10.5% of total adults in this age group. More than 80% of people with diabetes live in low- and middle-income countries. According to the aforementioned report, Africa is home to 23.6 million diabetes cases, which is the least compared to other continents. However, this number is expected to more than double by 2045. In Ethiopia, a similar report revealed that the overall prevalence of DM in adults was estimated to be 3.3% [1]. This magnitude is greater than the prevalence that had been reported in previous reports.

Due to the chronic nature of the disease, the majority of patients receive ambulatory care daily. This calls for encouraging and strengthening the self-care behaviors of all diabetes patients. Diabetes patients serve as their own primary care providers [5,6]. Evidence suggests that patients’ understanding of the diseases, proper self-care practices, and dedication to take part in disease management are crucial in getting the intended treatment outcome [6-8]. Diabetes self-care practice depends on patients’ understanding of both self-care practice and the disease condition as a whole. As literature from Ghana explained, patients´ knowledge about the disease had a direct impact on how often they exercise self-care [9]. Likewise, cross-sectional research undertaken in Ethiopia depicts a direct correlation between patients´ self-care practices and knowledge about the disease [10,11].

Despite the numerous studies conducted to examine the extent of self-care practice and associated factors, there is scant evidence of diabetes self-care knowledge and determinant factors in Ethiopia. Thus, this study aimed to bridge the gap by assessing the magnitude of diabetes self-care knowledge and driving factors among type II diabetes patients in North Central Ethiopia.

## Methods

**Study design, area, and period:** a facility-based cross-sectional study design was employed. The study was conducted in Shewa Robit and Kewot District, North Central Ethiopia, from March 22, 2022, to April 30, 2022.

**Study population:** the source population was all adult patients with diabetes who had regular outpatient follow-up in the selected health facilities at Shewa Robit Town and Kewet District in North Central Ethiopia.

**Eligibility criteria:** adult diabetic patients who had regular follow-up during the study period were included in the study. Patients who were critically ill and had hearing impairment were excluded from the study.

**Sample size determination and sampling procedure:** the sample size was estimated using the single population proportion formula, considering the prevalence of diabetes patients´ self-care knowledge (70.4%), which was taken from a study conducted in Ayder Comprehensive and Specialized Hospital in North Ethiopia [7] with the assumptions of a 95% confidence interval, 1.96 standard normal variable (Z-score), 5% margin of error, and adjusting 10% of non-response rate, the final sample size was 355.

Eight private and public health facilities were found in the study area. Four of them were selected by employing a simple random sampling approach. To allocate proportionally to the size, the total patient flows of each health facility were identified from the patient registration charts. Eventually, every fifth patient was selected for interview using a systematic sampling procedure. The first patient was chosen using a simple random sampling technique.

### Variables

**Dependent variable:** level of self-care knowledge of diabetes patients.

**Independent variables:** socio-demographic factors like age, sex, religion, educational status, marital status, and monthly income. In addition, clinical and healthcare-related factors include duration of diagnosis, family history, and type of DM, received health education, and source of health information.

**Operational definitions:** self-care knowledge of diabetic patients who responded greater than 50% was considered as having good knowledge. Whereas respondents who scored 50% or less were considered to have poor knowledge [12].

**Data collection instrument:** the data were collected by using a pre-tested, validated, and standardized questionnaire through face-to-face interviews. To maintain the validity of the tool, the questionnaire was adopted from the standard and translated into Amharic, and to verify consistency, translated back to English. The instrument comprised three sections: sociodemographic, health-related characteristics, and information source, and Diabetic Self-Care Knowledge Questionnaire (DSCKQ-30) item questions.

**Data collection procedure:** prior to collecting the data, briefly explained to the respondents the overall purpose of the study. In the end, written informed consent was secured from those who voluntarily participated in the study. Sociodemographic and knowledge-related data were collected by interviewing the study participants. Whereas clinical-related data were collected by reviewing patients´ charts.

**Data quality assurance:** the tool was originally developed in English, translated into Amharic, and back-translated into English to check consistency by different translators. To ensure the quality, three diploma-holder nurses for data collection and one BSc-holder health office were recruited and trained for two days about the general objective of the study, data collection system, and supervision process. Additionally, a pretest of the tool was conducted around 18 diabetic patients from Ataye Town in neighboring districts to the study site to check clarity, understandability, completeness, reliability, and consistency, and necessary revisions were made. Eventually, the principal investigator and supervisor checked the collected data for completeness, and corrective measures were taken accordingly.

**Data management and analysis:** data were entered into EpiData version 4.6 and transferred into SPSS version 23 for statistical analysis. Frequency tabulation was undertaken to identify inconsistencies and missing values prior to running any analysis. Data distribution was checked using the Shapiro-Wilk normality test. Categorical data were presented using descriptive statistics, frequency, and percentage. Whereas continuous variables were presented as mean ± SD. A binary logistic regression model was used to identify potential predictor variables for diabetic self-care knowledge. Variables with a p-value of less than 0.2 in the binary logistic regression analysis were entered into the multivariable logistic regression analysis. Then Adjusted Odds Ratio (AOR) with 95% CI and p-value < 0.05 were used to identify factors significantly associated with diabetic self-care knowledge.

**Ethical approval and consent form:** this study was undertaken in compliance with the guiding principles of the Declaration of Helsinki. An ethical clearance letter was granted from the institutional review committee of the College of Medicine and Health Science of Debre Berhan University. A permission letter was secured from Shewa Robit Town and Kewot District Health Office and respective health institutions. Prior to commencing the actual data collection, the purpose of the study and the rights of participants were explained to the study subjects. Written informed consent was obtained from the study participants, and the respondents were told that the information obtained from them would be kept with full confidentiality.

## Results

**Socio-demographic characteristics of participants:** from 355 study participants, 340 of them have responded, making a response rate of 95.8%. The majority of the study participants, 267 (77.1%), were urban residents. And one hundred twenty-seven of the participants (37.4%) were 50 years old and up. More than half (65%) of the research participants were male, and 247 (72.6%) of the respondents were married. About 190 (55.9%) of the study participants were orthodox religious followers. In terms of educational attainment, 127 (37.4%) of the participants had a college diploma or above. Around 156 (46.4%) of the participants were government employees, and 155 (45.6%) of the respondents had a monthly income of less than 1000 birr (Table 1).

**Table 1:** socio-demographic characteristics of diabetic patients at health facilities in Shewa Robit Town and Kewet District, North-Central, Ethiopia, 2021 (n=340)

Variable	Categories	N (%)
Place of residence	Urban	262(77.1)
	Rural	78 (22.9)
Age in years	Less than 30	28 (8.2)
	30 to 39	76 (22.4)
	40 to 49	109 (32.1)
	50 and above	127 (37.4)
Sex	Male	221 (65.0)
	Female	119 (35.0)
Marital status	Single	44 (12.9)
	Married	247 (72.6)
	Divorced	28 (8.2)
	Separated	7 (2.1)
	Windowed	14 (4.1)
Religion	Orthodox	190 (55.9)
	Protestant	116 (34.1)
	Muslim	30 (8.8)
	Catholic	4 (1.2)
Educational status	Can’t read or write	82 (24.1)
	Read and write only	42 (12.4)
	Primary school	50 (14.7)
	Secondary school	39 (11.5)
	College diploma and above	127 (37.4)
Occupation	Gov’t employee	156 (46.4)
	House wife	52 (15.3)
	Private employee	54 (15.9)
	Merchant	20 (5.9)
	Farmer	58 (17.1)
Monthly income	Less than 1000 birr	155 (45.6)
	1000-2000 birr	88 (25.9)
	Above 2000 birr	97 (28.5)

**Clinical characteristics of participants:** over half of the individuals in the research, 199 (58.5%), had diabetes for fewer than five years. Among the participants, the majority (254 or 74.4%) of the respondents had regular follow-up in diabetic clinics, and three-fourths (75.3%) of participants had received family care. The highest number of respondents, 265 (77.9%), had ever received diabetic-related health education. Of those, 174 (65.7%) of respondents had received health education from health professionals.

**Participants’ self-care knowledge:** in this research, more than half, 186 (54.7%) of the study participants had good knowledge of diabetes self-care. Correspondingly, overall self-care knowledge of respondents on modifiable lifestyles, adherence to diabetes self-care, and consequences of uncontrolled blood glucose level was 58.1%, 57.5%, and 60.4%, respectively (Table 2, Table 3, Table 4).

**Table 2:** diabetes self-care knowledge modifiable lifestyle items of diabetic patients at health facilities in Shewa Robit Town and Kewet District, North-Central, Ethiopia, 2021

Questions	Responses		Correctness
	Yes	No	
The fasting blood sugar (FBS) test can be used to monitor 2 to 3 months of blood sugar control	117 (34.4%)	223 (65.6%)	223 (65.6%)
Only the doctors should make plans on how a person with diabetes can achieve his/her target goals	135 (39.7%)	205 (60.3%)	205 (60.3%)
Blood glucose level should be measured before and after every planned physical activity	204 (60.0%)	136 (40.0%)	204 (60.0%)
Having physical activity for 20-30 minutes per session at least 3 days per week is essential (example of physical activities: brisk walking, house activities, climbing stairs)	192 (56.5%)	148 (43.5%)	192 (56.5%)
Regular exercise does not reduce the need for insulin or other diabetic drugs	142 (41.8%)	198 (58.2%)	198 (58.2%)
Maintaining a healthy weight is not important in the management of diabetes	166 (48.8%)	174 (51.2%)	174 (51.2%)
A person with diabetes should only ask for help when he/she feel sick from his/her healthcare team	147 (43.2%)	193 (56.8%)	193 (56.8%)
Cigarette smoking can worsen diabetes disease	188 (55.3%)	152 (44.7%)	188 (55.3%)
At the initiation of insulin therapy for a person with diabetes who may require it, appropriate advice on Self-monitoring blood glucose (SMBG) and diets should be given to the person	207 (60.9%)	133 (39.1%)	207 (60.9%)
There should be mutual agreement between a person with diabetes and the doctor if he/she cannot change a particular lifestyle and afford his/her drugs>	190 (55.9%)	150 (44.1%)	190 (55.9%)
A person with diabetes should take extra care of his/her feet, especially when cutting his/her toenails	213 (62.6%)	127 (37.4%)	213 (62.6%)
Tight elastic hose or socks are not bad for a person with diabetes	160 (47.1%)	180 (52.9%)	180 (52.9%)
A person with diabetes should take care of his/her teeth, brush and floss his/her teeth every day	213 (62.6%)	127 (37.4%)	213 (62.6%)
No person should check the blood sugar and blood pressure of a diabetic patient except a qualified medical doctor and other health personnel in the hospital	186 (54.7%)	154 (45.3%)	186 (54.7%)
A person with diabetes should report any change in his eyesight to his doctor	204 (60.0%)	136 (40.0%)	204 (60.0%)
SMGB allows doctors and other healthcare teams together data for treatment planning	190 (55.9%)	150 (44.1%)	190 (55.9%)
SMGB enables a person with diabetes to monitor and react to changes in his/her blood sugar levels	210 (61.8%)	130 (38.2%)	210 (61.8%)
Monitoring blood pressure is not as important as monitoring blood glucose in a person with diabetes	153 (45.0%)	187 (55.0%)	187 (55.0%)
Overall correctness			58.1%

**Table 3:** diabetes self-care knowledge adherence to self-care practice items of diabetic patients at health facilities in Shewa Robit Town and Kewot District, North-Central, Ethiopia, 2021

Questions	Responses		Correctness
	Yes	No	
Dietary instructions should be written out, even if the person with diabetes is illiterate: someone at home should be available to interpret it for him/her	137 (40.3%)	203 (59.7%)	203 (59.7%)
A person with diabetes taking diabetic medicines even when he/she feel good is a waste of money	144 (42.4%)	196 (57.6%)	196 (57.6%)
Being drunk while on diabetic drugs is not a serious problem	148 (43.5%)	192 (56.5%)	192 (56.5%)
Diet and exercise are not as important as medication in controlling diabetes	153 (45.0%)	187 (55.0%)	187 (55.0%)
Instructions about drugs and other self-care practices must not be strictly followed	135 (39.7%)	205 (60.3%)	205 (60.3%)
Regular medical checkups are not essential when a person with diabetes is feeling well	165 (48.5%)	175 (51.5%)	175 (51.5%)
Taking a low-dose Aspirin tablet every day decreases the risk of having a heart attack and stroke	210 (61.8%)	130 (38.2%)	210 (61.8%)
Diabetes Drugs are not taken throughout the lifetime of a person with diabetes	145 (42.6%)	195 (57.4%)	195 (57.4%)
Overall correctness			57.5%

**Table 4:** knowledge level of adherence to self-care practice items of diabetic patients at health facilities in Shewa Robit Town and Kewet District, North Shoa Zone, Amhara Region, Ethiopia, 2021

Questions	Responses		Correctness
	Yes	No	
If blood sugar is close to normal, a person with diabetes is likely to have more energy, feel less thirsty, and urinate less often	205 (60.3%)	135 (39.7%)	205 (60.3%)
Shaking, confusion, behavioral changes, and sweating are signs of high blood sugar	214 (62.9%)	126 (37.1%)	214 (62.9%)
Prolonged high blood sugar levels can cause eye problems or even blindness	235 (69.1%)	105 (30.9%)	235 (69.1%)
Prolonged uncontrolled blood sugar levels can cause heart attack, stroke, and kidney problems	167 (49.1%)	173 (50.9%)	167 (49.1%)
Overall correctness			60.4%

**Factors associated with diabetic self-care knowledge:** a binary logistic regression was undertaken to identify potential predictor variables that affect diabetic self-care knowledge. Variables with a p-value of 0.2 or less in binary logistic regression were fitted into a multivariate logistic regression model. In this study, variables that showed a significant association were educational status, monthly income, regular diabetes follow-up, and having ever been in diabetes health education found to have a significant association with good self-care knowledge.

Respondents with a diploma and above educational level were 3 times more likely to have good self-care knowledge compared to those who cannot read and write (AOR=3.407; 95% CI=1.337, 8.684). Study participants, who had a monthly income above 2,000 birr, were 4 times more likely to have good self-care knowledge than those who had a monthly income below 1,000 birr (AOR= 4.384; 95% CI=1.548, 12.411). Respondents who had regular follow-up were 9 times more likely to have good self-care knowledge compared to those who didn´t have regular follow-up (AOR=8.910; 95% CI=2.903, 17.349). Ever being in diabetic health education revealed a consistent significant association in both bi-variable and multivariable analysis; those who had ever been in diabetic health education were 5 times more likely to have good self-care knowledge than those who had never been in diabetic health education (AOR=5.554; 95% CI=1.820, 16.953) (Table 5).

**Table 5:** factors associated with good self-care knowledge

Variables	Category	Knowledge level		AOR (95% CI)
		Good N (%)	Poor N (%)	
Place of residence	Urban	172 (65.6%)	90 (34.4%)	1
	Rural	13 (16.9%)	64 (83.1%)	102 (0.33- 3.12)
Sex	Male	135 (61.1%)	86 (38.9%)	1
	Female	51 (42.9%)	68 (57.1%)	0.85 (0.41-1.75)
Religion	Orthodox	83 (43.7%)	107 (56.3%)	1
	Protestant	92 (79.3%)	24 (20.7%)	2.50 (0.25-5.00)
	Muslim	10 (33.3%)	20 (66.7%)	0.34 (0.11-1.01)
	Catholic	1 (25.0%)	3 (75.0%)	0.14 (0.01-1.80)
Educational Level	Can’t read or write	18 (22.0%)	64 (78.0%)	1
	Read and write only	15 (35.7%)	27 (64.3%)	0.48 (0.15-1.53)
	Primary school	25 (50.0%)	25 (50.0%)	0.67 (0.18-2.49)
	Secondary school	26 (66.7%)	13 (33.3%)	1.31 (0.32-5.30)
	College diploma and above	102 (80.3%)	25 (19.7%)	3.40 (1.33-8.68) *
Occupation	Farmer	12 (20.7%)	46 (79.3%)	1
	Government employee	118 (75.6%)	38 (24.4%)	0.88 (0.19-4.09)
	Housewife	18 (34.6%)	34 (65.4%)	0.73 (0.15-3.40)
	Private employee	27 (50.0%)	27 (50.0%)	1.17 (0.19-7.06)
	Merchant	11 (55.0%)	9 (45.0%)	1.86 (0.47-7.30)
Monthly income	Less than 1000 birr	50 (32.3%)	105 (67.7%)	1
	1000 to 2000 birr	53 (60.2%)	35 (39.8%)	1.01 (0.43-2.34)
	Above 2000 birr	82 (85.4%)	14 (14.6%)	4.38 (1.54-12.41) *
Regular DM follow-up	No	6 (7.1%)	79 (92.9%)	1
	Yes	180 (70.6%)	75 (29.4%)	8.91 (2.90-17.34) *
Support from family	No	21 (25.0%)	63 (75.0%)	1
	Yes	165 (64.5%)	91 (35.5%)	1.51 (0.66-3.45)
Ever been in health education about DM	No	7 (9.3%)	68 (90.7%)	1
	Yes	179 (67.5%)	86 (32.5%)	5.55 (1.82-16.95) *

AOR: adjusted odds ratio; CI: confidence interval; *: associations are significant at P < 0.05; DM: diabetes mellitus

## Discussion

Diabetes is a chronic and complex metabolic disorder with multiple long-term and short-term complications. In order to maintain the desired outcomes of treatment and quality of life, patients´ involvement in the management plays a prominent role. Hence, assessment of the patient´s self-care knowledge is an important first step in designing an appropriate diabetes education program. Therefore, this study was conducted with 340 participants to assess their level of knowledge towards diabetes self-care. This study found that one out of two DM patients has good knowledge of diabetes self-care. However, our finding is relatively lower than the study conducted in North Shoa, Oromia region of Ethiopia [13]. Mekele Ayder Comprehensive Hospital in North Ethiopia [7], Jimma Medical Center, Southwest Ethiopia [14], and Arsi, Zone Southeast, Ethiopia [15] which were 67.8%, 70.4%, 63.3%, and 64.8%, respectively. This discrepancy could be due to differences in the sociodemographic characteristics of the study participants. Furthermore, living closer to the capital city provides the chance to frequently access medical services.

This study also discovered that educational level, monthly income, regular follow-up and having ever had diabetic-related health education were factors significantly associated with good diabetic self-care knowledge. According to this study, participants who had a diploma and higher educational level were three times more likely to have better diabetes self-care knowledge than those who could not read and write. This finding agrees with a cross-sectional study conducted at Jimma Medical Center (JMC), Ethiopia [14]. A study from Nigeria, Thailand, and Brazil also revealed that those who had a higher academic level had better self-care knowledge than their counterparts [8, 16, 17]. It may not be surprising that knowledge is acquired through education. Moreover, individuals who have a higher academic level have a greater chance of obtaining knowledge from various sources, like mass media, reading materials like books, and the internet.

The present study discloses that an individual who earns a monthly income of greater than 2000 birr is four times more likely to have good knowledge of diabetes self-care as compared to those who earn a monthly income of less than 1000 birr. This discovery is consistent with findings recorded in other studies. A cross-sectional study in Nigeria indicates that participants with higher income have good knowledge of diabetes self-care [8]. The possible justification could be that making a higher monthly income could open up the opportunity to access relevant information about diabetes self-care.

According to the findings, participants who had regular follow-up were nine times more likely to have diabetes self-care knowledge as compared to those who did not have regular follow-up. Despite the fact that there is little evidence to compare our findings with others, several studies have shown that diabetes patients with a longer duration of disease and regular follow-up had better knowledge about the disease and its complications than newly diagnosed patients. A cross-sectional study from Northwest Ethiopia showed that patients with a longer duration had better knowledge than their counter group [18]. Similarly, research from Benin and Sri Lanka revealed that study subjects with longer duration and regular follow-up had better knowledge about the disease and complications due to poor management [19,20]. On the contrary, a study from Kuwait indicates that patients with a lower duration of disease had a lower level of knowledge regarding the disease and were less likely to comply with treatment recommendations [21]. This is partly explained because these patients gained better and up-to-date information from health professionals and peers regarding diabetes mellitus.

This study demonstrated that patients who had ever received health education about diabetes had better knowledge regarding diabetes self-care than those who had not attended diabetes health education. This finding is in line with a cross-sectional study conducted in North Shewa, Ethiopia, and South Western Uganda [13,22]. Apart from these observational studies, trial studies from Nigeria and Bangladesh proved that precise and patient-friendly diabetes health education enhances their knowledge about self-care and the disease overall [23,24]. This similarity might be explained that health education is the fundamental way to acquire knowledge. Hence, patients who have had previous exposure to diabetic health education have a better understanding of self-care than those who do not have diabetes-related health education experience.

**Limitations of the study:** despite this study having some strengths, this study has several limitations. A cross-sectional study was employed to undertake this investigation. It does not provide the ability to infer temporal relations. The use of the mean score to dichotomize the score obtained from the tool can be considered a limitation. However, we were forced to use it due to the absence of a suggested cut-off point.

## Conclusion

The finding from this study discloses that more than half of the study participants had good knowledge regarding diabetes self-care. Furthermore, this study suggests that participants with post-secondary education, better monthly income, and attending diabetes health education are important predictors of good diabetes self-care knowledge.

***What is known about this topic***

- *Self-management knowledge helps to ensure optimum glycemic control;*
- *Diabetes health education is an integral part of clinical management of DM.*

***What this study adds***

- *More than half of the study participants in North-Central Ethiopia had better knowledge about diabetes self-care knowledge;*
- *Socioeconomic and behavior change communication-related factors significantly explain diabetes patients´ self-care knowledge.*
